# Surgical Treatment of Posttraumatic Radioulnar Synostosis

**DOI:** 10.1155/2016/5956304

**Published:** 2016-02-08

**Authors:** S. Pfanner, P. Bigazzi, C. Casini, C. De Angelis, M. Ceruso

**Affiliations:** Hand Surgery and Reconstructive Microsurgery Unit, AOU Careggi, 50139 Florence, Italy

## Abstract

Radioulnar synostosis is a rare complication of forearm fractures. The formation of a bony bridge induces functional disability due to limitation of the pronosupination. Although the etiology of posttraumatic synostosis is unknown, it seems that the incidence is higher in patients who have suffered a concomitant neurological or burn trauma, and extensive soft tissue injury, mainly due to high-energy impact. Surgical treatment, such as reinsertion of distal biceps tendon into the radius, seems to be another possible factor. The aim of the surgical treatment is to remove the bony bridge and restore complete range of movement (ROM), thus preventing recurrence. Literature does not indicate a preferred type of surgical procedure for the aforementioned complication; however, it has been shown that surgical interposition of inert material reduces the formation rate of recurrent bony bridge. We describe a surgical technique in two cases in which the radius and ulna were wrapped with allogenic, cadaver fascia lata graft to prevent bony bridge formation. The data from 2 years of follow-up are reported, indicating full restoration of ROM and no recurrence of synostosis.

## 1. Introduction

Posttraumatic formation of a radioulnar bony bridge synostosis is a rare but serious complication that can develop in a bony ankylosis with a complete limitation of pronosupination [1]. The main risk factors are high-energy trauma, Monteggia's-like type of fracture, both bone forearm fractures at the same level, comminuted fractures with soft tissue compression, and free bone fracture fragments included in the interosseous membrane [2–4]. Some surgical conditions can also favor the formation of these bony bridges, such as delayed surgical treatment, a single approach (Boyd approach), excessively long cortical screws, prolonged immobilization, and delayed rehabilitation. Literature reports the incidence of posttraumatic synostosis ranging from 0 to 9.4%, occurring more frequently after road accidents and high-energy sports injuries [2, 5]. Signs and symptoms include reduction of pronosupination, both active and passive, usually without pain, and occasionally associated with a reduction of range of movement of the radiocarpal joint [1, 3, 6]. Anteroposterior and laterolateral X-ray images show single or multiple bony bridges. Vince and Miller's classification is the most commonly used. It divides posttraumatic synostosis into three types: type 1 involving the distal 1/3 of the forearm, type 2 involving the median 1/3, and type 3 involving the proximal 1/3 [2]. Type 2 is the most common type and is associated with the best prognosis [1]. Literature indicates that the aim of surgical treatment is to restore complete range of motion by removing bony bridges; however, there are no clear-cut guidelines regarding the treatment for prevention of recurrence [3]. Appropriate surgical timing seems to be the single most important factor in preventing recurrences, and early surgical treatment is more likely to induce recurrence of bony bridges. Literature indicates optimal timing to be from a minimum of 6 months up to a maximum of two years after the trauma [1, 2]. The type of treatment depends mainly on the location of the bony bridge [4]. Synostosis type 1 can be treated with Sauve-Kapandji procedure if the distal radioulnar joint is affected by degenerative process and the bony bridge is under the pronator quadratus muscle. In type 2 synostosis, the most appropriate treatment seems to be the excision of the bony bridge and interposition of inert material. Such treatment is also indicated for synostosis located at the level of the bicipital tuberosity. Type 3 synostoses are further divided into three different subgroups: type 3A, which affects the proximal third of the forearm without involving the articular surface and is treated as type 2; type 3B, where only the radioulnar joint involved is treated with the excision of the radial head; type 3C, where the radiohumeral joint is involved and, consequently, the treatment is an arthroplasty [4, 5]. Review of literature indicates different surgical approaches for preventing the formation of recurrent synostosis: (1) interposition of inert, synthetic material, upon bony bridge excision, band, or fat graft [2, 3, 7–9]; (2) nonsteroidal anti-inflammatory drugs (NSAID), including indomethacin, ibuprofen, tenoxicam, naproxen, flurbiprofen, ketorolac, and diclofenac, which were found to be effective in preventing the formation of exostoses, while the same was not found for bisphosphonates [3, 10]; (3) low-dose radiation treatment (it does not find agreement in the literature regarding its utility for this specific indication, although it was proved effective in preventing the formation of heterotopic hip arthroplasty ossifications [4–6]).

Literature indicates that rehabilitation treatment is extremely important and should be started early and be frequent. Some authors propose the use of splints in maximum pronation and supination during the transition from passive to active mobilization. The possibility of overall recurrence for all types of synostosis has been assessed to be around 29%, but those of type 2 vary between 0% and 5% [2, 5] in relation to various types of surgical treatment.

## 2. Case Report

**Figure 1:**
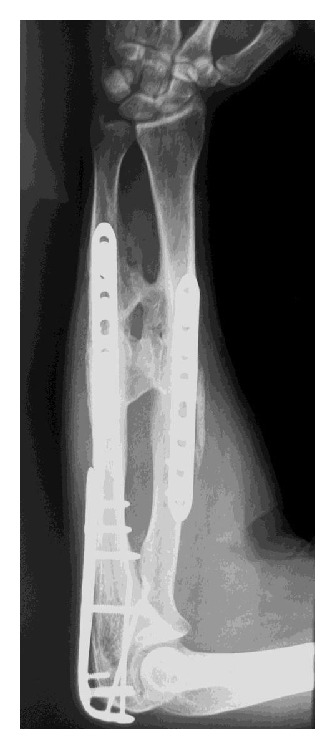
Right forearm in a 39-year-old man with a radioulnar synostosis type 2 18 months after the first surgery procedure for reduction and fixation of the fractures.

In two cases of radioulnar posttraumatic synostosis, an excision of radioulnar synostosis and interposition of cadaveric fascia lata graft was performed. Both patients were males and at the time of surgery were 39 and 24 years old. The older patient suffered a type 2 synostosis and was operated on 18 months after the initial surgery for reduction and internal fixation of the fracture, involving both radius and ulna bones of the forearm at the same level and a concomitant fracture of olecranon (Figure 1), occurring after a motorcycle road accident.

**Figure 2:**
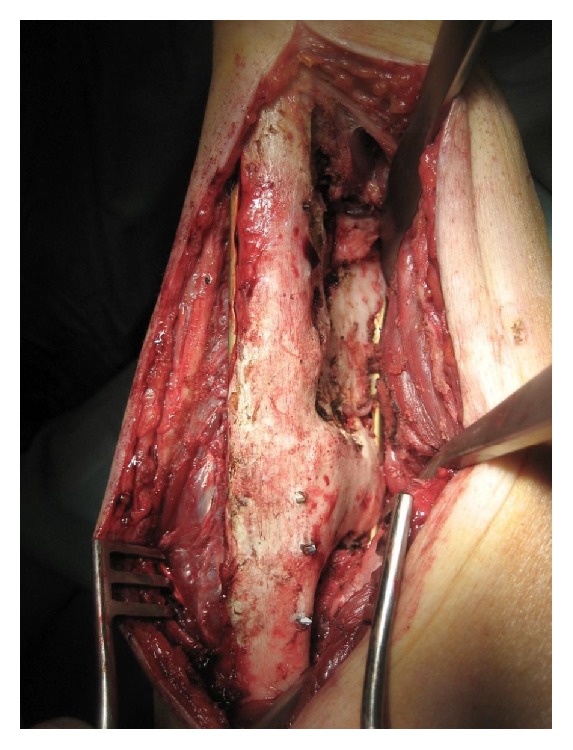
The intraoperative view shows a bone ankylotic bridge of 10 cm length and 2 cm thickness.

**Figure 3:**
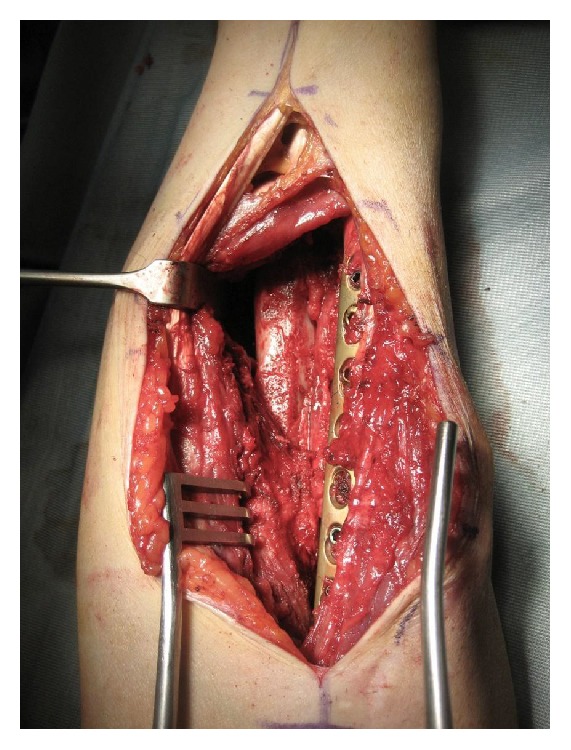
Complete removal of the synostosis.

**Figure 4:**
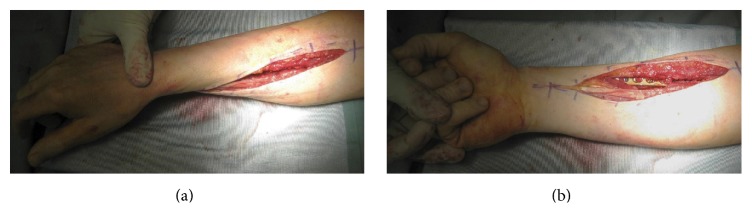
The pronosupination was complete immediately after excision and tested intraoperatively.

**Figure 5:**
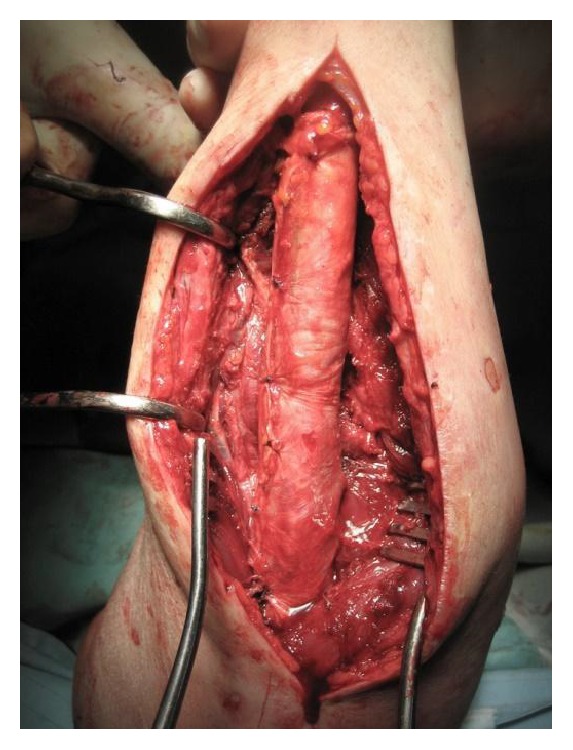
Allogenic cadaver graft of fascia lata was double-wrapped around the ulna and sutured on itself.

**Figure 6:**
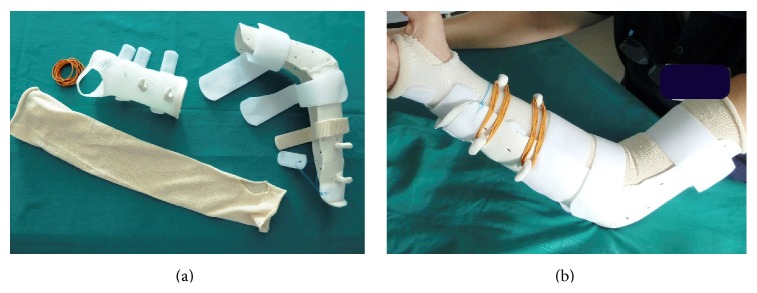
The custom-made dynamic splint works in the first month to act in static progressive mobilization and from the second month starts an active mobilization.

**Figure 7:**
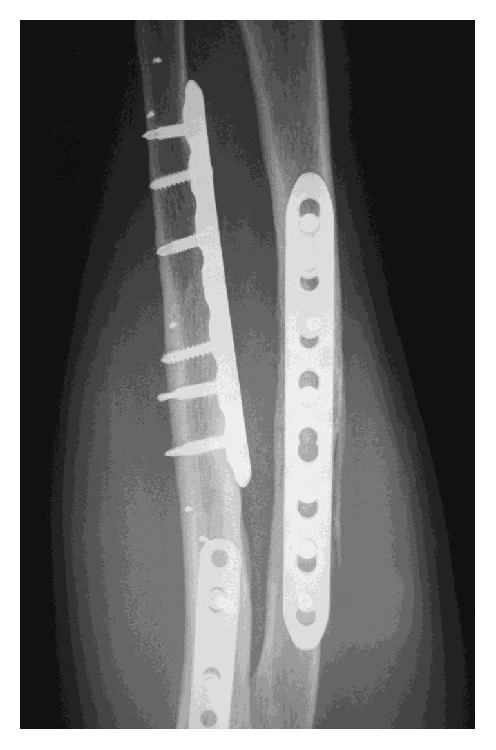
X-ray follow-up at 12 months after second surgery; there is no evidence of recurrence of bone formation.

**Figure 8:**
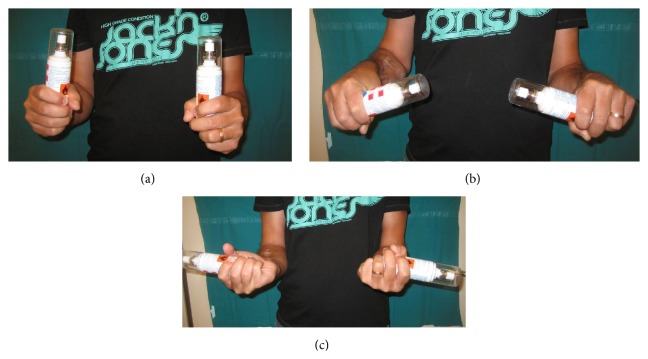
Clinical results at 1 year after excision of the synostosis: complete recovery of ROM in pronosupination.

The second patient, who had a type 3A synostosis, was encountered as result of a fracture of both bones of the forearm at the same level after a motorcycle accident that had been treated by initial surgery 20 months before, performing internal fixation by plates. In both cases, the fractures were caused by high-energy trauma and showed large soft tissue damage and the involvement of the interosseous membrane. Both patients also suffered additional multiple injuries and neurological damage with transient comatose state and required several days in the intensive care unit (ICU). Consequently, mobilization was extremely delayed. Intra- and post-ICU serial X-ray evaluation disclosed, for both cases, progressively growing bony bridge synostosis. Once the patients were clinically stable and ready for surgery, a CT imaging of the synostosis was also performed. The time between the initial surgical treatment of fractures and the removal of synostosis was, respectively, 18 months and 20 months. The surgical approach was done using the previous surgical incision. While taking care to protect the vascular and nervous structures, we exposed the synostosis, which in the first case had a length of about 10 cm and thickness of about 2 cm (Figure 2), while in the second case it had a length of 6 cm and a thickness of 3 cm. In both cases, complete excision of the synostosis was performed, while in the second case it was also necessary to remove the radial plate. After the removal of the synostosis (Figure 3), the range of movement of pronosupination for both patients was tested and shown to be complete (Figure 4). Allogenic cadaver graft of fascia lata 5 cm wide and 12 cm long was double-wrapped around the ulna in the first case and around the radius in the second case and was anchored to the bone by suture anchors (mini-mitek 2/0). At the end, the graft was sutured to itself (Figure 5). Both patients initiated immediate treatment with etoricoxib selective COX-2 (Celebrex 200 mg, Tauxib 90 mg) for 2 months and, after an initial period of immobility of around ten days to allow proper healing of the wound, were subjected to intense rehabilitation treatment and the use of splinting in alternating maximum pronation or supination positions, in order to restore the full range of movement (Figure 6). The rehabilitation treatment started, for both patients, on the third postop day, so as to avoid possible wound bleeding. The cast was splint-open to permit painless range of passive rehabilitation movements. Ten pronation-supination and flexion-extension exercises of the elbow were repeated 3 to 4 times a day during the first postop week. Ice packs were applied for 15 minutes after the exercises and before repositioning of the cast. At the tenth postop day, the cast was definitively removed and substituted with a custom-made dynamic splint. Initially, apart from frequent rehabilitation exercises, the splint was customized to act in static progressive mode. In both patients, the elastic bandage application for edema containment and improved lymphatic return was initially light, becoming progressively more forceful. The aforementioned passive mobilization and customized splinting permitted the patients to regain 70% of the range of movement during the first month, while during the second postop month, where active mobilization was applied, another 20 and 25 degrees of ROM were gained for the first and the second patient, respectively. The splint was applied at nighttime for 3 consecutive months, alternating supination and pronation positioning, which resulted in significant improvement of passive muscle stretching. Throughout treatment, the pain values, as reported by the patients, never surpassed 2 on the Visual Analogic Scale. At 12- and 24-month, clinical and X-ray follow-up for the first (Figure 7) and second case, respectively, patients showed a complete recovery of the range of motion in pronosupination (Figure 8). Pain was absent, and normal full daily activities were reported. X-ray imaging did not show any recurrence of synostosis. The pronosupination was complete immediately after surgery and, apart from the initial two weeks, range of motion remained complete until the final follow-up.

## 3. Discussion

Heterotopic ossification most frequently occurs in the upper extremity after high-energy injury, resulting in severe functional impairment. Currently, there is no agreement on the golden standard of surgical treatment and the type of postoperative adjuvant treatment in terms of drug use or low dosage radiotherapy. In any case, after the removal of the synostosis, the interposition of inert material is recommended in the literature to prevent recurrence. The role of postoperative protracted (up to six months) and intense rehabilitation treatment is fundamental. Although our experience is limited to two cases, bony bridge excision and fascia lata interposition show excellent results and should be considered sufficient for the prevention of the formation of new bone coalition over time.

## References

[1] Dohn P., Khiami F., Rolland E., Goubier J.-N. (2012). Adult post-traumatic radioulnar synostosis. *Orthopaedics and Traumatology: Surgery and Research*.

[2] Vince K. G., Miller J. E. (1987). Cross union complicating fracture of the forearm. *The Journal of Bone & Joint Surgery—American Volume*.

[3] Friedrich J. B., Hanel D. P., Chilcote H., Katolik L. I. (2006). Use of tensor fascia lata interposition grafts for the treatment of posttraumatic radioulnar synostosis. *Journal of Hand Surgery*.

[4] Hastings H., Graham T. J. (1994). The classification and treatment of heterotopic ossification about the elbow and forearm. *Hand Clinics*.

[5] Jupiter J. B., Ring D. (1998). Operative treatment of post-traumatic proximal radioulnar synostosis. *The Journal of Bone & Joint Surgery—American Volume*.

[6] Cullen J. P., Pellegrini V. D., Miller R. J., Jones J. A. (1994). Treatment of traumatic radioulnar synostosis by excision and postoperative low-dose irradiation. *Journal of Hand Surgery*.

[7] Proubasta I. R., Lluch A. (1995). Proximal radio-ulnar synostosis treated by interpositional silicone arthroplasty—a case report. *International Orthopaedics*.

[8] Lytle I. F., Chung K. C. (2009). Prevention of recurrent radioulnar heterotopic ossification by combined Indomethacin and dermal/silicone sheet implant: case report. *Journal of Hand Surgery*.

[9] Sonderegger J., Gidwani S., Ross M. (2011). Preventing recurrence of radioulnar synostosis with pedicled adipofascial flaps. *The Journal of Hand Surgery: European Volume*.

[10] Thomas B. J., Amstutz H. C. (1985). Results of the administration of diphosphonate for the prevention of heterotopic ossification after total hip arthroplasty. *The Journal of Bone & Joint Surgery—American Volume*.

